# Negative Affectivity Predicts Lower Quality of Life and Metabolic Control in Type 2 Diabetes Patients: A Structural Equation Modeling Approach

**DOI:** 10.3389/fpsyg.2017.00831

**Published:** 2017-05-24

**Authors:** Chiara Conti, Giulia Di Francesco, Lara Fontanella, Danilo Carrozzino, Chiara Patierno, Ester Vitacolonna, Mario Fulcheri

**Affiliations:** ^1^Department of Psychological, Health, and Territorial Sciences, University “G. d'Annunzio” of Chieti-PescaraChieti, Italy; ^2^Department of Legal and Social Sciences, University “G. d'Annunzio” of Chieti-PescaraChieti, Italy; ^3^Psychiatric Research Unit, Mental Health Centre North Zealand, University of CopenhagenHillerød, Denmark; ^4^Department of Medicine and Aging, University “G. d'Annunzio” of Chieti-PescaraChieti, Italy

**Keywords:** Type D personality, structural equation modeling, metabolic control, negative affectivity, social inhibition, quality of life, diabetes mellitus

## Abstract

**Introduction:** It is essential to consider the clinical assessment of psychological aspects in patients with Diabetes Mellitus (DM), in order to prevent potentially adverse self-management care behaviors leading to diabetes-related complications, including declining levels of Quality of Life (QoL) and negative metabolic control.

**Purpose**: In the framework of Structural Equation Modeling (SEM), the specific aim of this study is to evaluate the influence of distressed personality factors as Negative Affectivity (NA) and Social Inhibition (SI) on diabetes-related clinical variables (i.e., QoL and glycemic control).

**Methods:** The total sample consists of a clinical sample, including 159 outpatients with Type 2 Diabetes Mellitus (T2DM), and a control group composed of 102 healthy respondents. All participants completed the following self- rating scales: The Type D Scale (DS14) and the World Health Organization QoL Scale (WHOQOLBREF). Furthermore, the participants of the clinical group were assessed for HbA1c, disease duration, and BMI. The observed covariates were BMI, gender, and disease duration, while HbA1c was considered an observed variable.

**Results:** SEM analysis revealed significant differences between groups in regards to the latent construct of NA and the Environmental dimension of QoL. For the clinical sample, SEM showed that NA had a negative impact on both QoL dimensions and metabolic control.

**Conclusions:** Clinical interventions aiming to improve medication adherence in patients with T2DM should include the psychological evaluation of Type D Personality traits, by focusing especially on its component of NA as a significant risk factor leading to negative health outcomes.

## Introduction

Diabetes Mellitus (DM) is a major cause of morbidity and mortality provoking a significant economic burden to the national health care system worldwide (Zhang et al., 2010). Furthermore, as also reported by patients, DM is a clinically significant source of both physical and psychological disability leading to diminishing levels of Quality of Life (Qol) (World Health Organization, 1980; Topp et al., 2015; Holmes-Truscott et al., 2016). In this regard, the study of DM comprises the analysis of relative contribution of psychological factors that significantly affect medical outcomes, such as medication adherence, a balanced diet and an appropriate exercise, and, when necessary, the regular monitoring of blood glucose levels (Cramer, 2004). Therefore, it is highly relevant from a diagnostic and treatment perspective to include psychological assessment in the clinical evaluation of DM, especially when considering that without patient's adherence to medications, there are many potential health risks for patients with DM such as: persistent sub-optimal levels of metabolic control, presence of diabetes-related complications, the deterioration of QoL, and increased health care utilization. In regards to glycemic control, it is also significantly associated with age, race/ethnicity, duration of DM, type and number of medications taken, psychological factors, obesity, and family support (van Dooren et al., 2016), although most studies have examined cross-sectional associations.

Thus, when focusing on the psychological factors that influence patients' adherence to treatments, several studies highlighted that psychological distress and negative mental health factors, such as depression (Egede and Dismuke, 2012; Roy and Lloyd, 2012) and anxiety (Smith et al., 2013), are common psychopathological symptoms among patients with DM. Indeed, a large body of studies found that the above reported psychological factors are associated with sub-optimal glycemic control (Lustman et al., 2000), DM complications (de Groot et al., 2001), and decreasing levels of QoL (Chew et al., 2015). The combination of DM with negative mental health aspects, mainly depression and anxiety, is associated with an impaired QoL, as well as with a greater difficulty in patients to self-monitor their blood glucose levels (Lewko et al., 2012). Lane et al. (2000) investigated the hypothesis, that personality traits may give new insight to alterations of metabolic control in patients with Type 2 DM(T2DM) undergoing standard management (Lane et al., 2000).

An emerging psychological risk factor in the cardiovascular research domain is Type D Personality, or Distressed personality, which is defined by the combination of two personality traits as follows: Negative Affectivity (NA) and Social Inhibition (SI) (Denollet, 2000). NA has also been conceptualized as neuroticism (McCrae and Costa, 1987; Eysenck and Furnham, 1993; Keogh and Reidy, 2000; Denollet, 2005) and its definition is based on the individual tendency to experience negative emotional states comprising dysphoria, feelings of tension and worry across time, and situations. Furthermore, the construct of NA arose from the original research literature on emotions and has previously been described as “a stable, heritable trait tendency to experience a broad range of negative feelings such as worry, anxiety, self-criticisms, and a negative self-view” (Denollet, 2005). The second component of Type D Personality is SI involving the inclination to inhibit the expression of emotions, thoughts, and behaviors in social interaction, due to concern of negative reactions from others (Denollet, 2000, 2005). The simultaneous presence of both traits is the extensively approved psychological characterization of the Type D Personality. Type D patients have an increased risk for a wide range of adverse health outcomes, including expressions of poor QoL in several medical settings. Suboptimal self-care behavior, such as poor medication adherence, is one potential mechanism through which Type D may exert a negative influence on health outcomes.

The Hemoglobin A1c (HbA1c) is a useful indicator, in order to study patients'retrospective glycemic control over a 2/3-month period. To date, very few studies have evaluated the relationship between Type D Personality and glycemic control, and none have found significant associations between Type D Personality and HbA1c. However, with respect to the association between levels of metabolic control and Type D Personality, Nefs et al. (2015) have found that Type D Personality was not related to HbA1c directly, but was associated with unhealthy behaviors and negative emotions that are likely to have harmful impact on DM clinical variables (Nefs et al., 2015). In a recent systematic review of literature (Conti et al., 2016), the authors have reported that Type D Personality was significantly associated with both negative medical outcomes (Denollet et al., 2010) and emotional distress (Nefs et al., 2012) in patients with DM. However, no studies have currently examined the potential role of Type D Personality on QoL in patients with DM.

Hence, the aim of our research study is to evaluate to what extent specific traits of Type D Personality, i.e., NA and SI, affect QoL and metabolic control (HbA1C) in patients with T2DM, by performing a specific statistical analysis defined as Structural Equation Modeling (SEM). Thus, our hypothesis was that NA and SI dimensions affect the QoL and HbA1c levels in a sample of patients with T2DM.

## Materials and methods

### Participants

The total sample consisted of 261 participants divided as follows: the clinical sample comprised 159 outpatients with T2DM and the control group 102 healthy respondents. The clinical sample, having a medically documented diagnosis of T2DM according to the diagnostic criteria of the WHO, attended this study from the Department of Diabetology at the University Clinical Hospital of Chieti, Italy. All patients were contacted by clinical psychologists in collaboration with medical diabetologists.

We included patients with T2DM of both sexes who met the following inclusion criteria: (a) adult outpatients with an age ranging from 35 to 80 years; (b) only outpatients medically treated at the University Hospital of Chieti; (c) outpatients who have been diagnosed with T2DM, previously or during the study period. By contrast, from the study were excluded patients who were exclusively attended by specialists due to other types of DM (e.g., T1DM), those having cognitive impairment or advanced disease, and those who were unable to perform or understand the self-rating scales.

The control sample included 102 participants from the Italian general population residing in the Central and Southern provinces of Italy. We included individuals of both sexes without DM and with an age ranging from 35 to 80 years. Individuals unable to perform or understand the self-rating scales, were excluded from the study. All participants completed the following self-rating scales: The Type D Scale (DS14), and the World Health Organization QoL Scale (WHOQOL-BREF). Furthermore, the participants of the clinical group were assessed for HbA1c, disease duration, and Body Mass Index (BMI).

### Ethics statement

The study was previously approved by the Head Physician of the Diabetes Medical Care Unit. Subsequently, the research protocol was also authorized by the President of the Master's Degree in Clinical and Health Psychology, Department of Psychological, Health, and Territorial Sciences, University “G. d'Annunzio” of Chieti-Pescara, Italy. The research was conducted with respect for the rights of all participants and the data were analyzed entirely anonymously. That is, rating scale evaluations, and HbA1c measurements were conducted as a part of everyday “real world” clinical practice assessment of patients, who fully anonymously filled out questionnaires. Moreover, each patient had to understand the research aims of the study and signed an informed written consent document. The same procedure was used for healthy participants, acting as controls, who were enrolled following informed and signed written consent. Thus, all participants were volunteers, who filled out the questionnaires in a confidential setting.

When taking into account that the original study only encompassed use of non-invasive and confidential patient self-rating scales, the study received approval by the Institutional Review Board of the University “G. d'Annunzio” of Chieti-Pescara, on the basis of the ethical principles for medical research involving human subjects developed according to the World Medical Association Declaration of Helsinki (Rickham, 1964) and its subsequent revisions.

### Measures

#### Demographic information

The demographic and socioeconomic factors such as age, gender, education level, employment status, marital status, and housing situation were collected at baseline.

#### Type D personality

We adopted the Italian short version of the Type D Scale-14 (DS14) consisting of 14 questions about personality, by using 14 items, 7 of which assessing the subcomponent NA and the remaining 7 evaluating SI with a 5-point Likert response scale ranging from 0 (false) to 4 (true). A pre-determined cut-off of ≥ 10 on both subscales was used to classify participants as Type D Personality (i.e., NA of ≥ 10 and SI of ≥ 10).

The DS14 is a valid instrument, with high internal consistency (Cronbach's α NA = 0.87; SI = 0.87) and good test-retest reliability (Denollet, 2005).

#### Quality of life

The Italian short version of the World Health Organization QoL Questionnaire (WHOQOL-BREF) was used to assess QoL (The WHOQOL Group, 1998a,b). This questionnaire was developed with 15 international field centers, in order to obtain an assessment tool that would be applicable cross-culturally. The four domains of the WHOQOL-BREF are physical health, psychological (e.g., self-esteem), social relationships (e.g., social support), and environment (e.g., freedom, physical safety). Subjects would rate all items on a 5-point Likert-type scale.

#### Clinical characteristics

At baseline, the clinical outcomes from standard care laboratory tests including HbA1c, BMI, the duration of DM, and medical complications (i.e., neuropathy, nephropathy, retinopathy, glaucoma, hypertension, other macro-, and micro-cardiovascular problems) were obtained from the patients' medical records.

#### Medication adherence

The Hemoglobin A1c (HbA1c) reflects the glycemic control over a 2/3-months period and provides an important indication of retrospective glycemic control.

### Statistical analysis

Data were analyzed using STATA 13, while descriptive statistics were used to assess the participant characteristics and the measured variables. Cronbach's α was computed for the items nested in each of the analyzed dimensions. SEM was used both to test differences between the control group and the clinical group, and to assess effects of the latent variables NA and SI on the QoL dimensions and HbA1c.

SEM is a set of statistical techniques used to measure and analyze the relationships of observed and latent variables. Similar but more powerful than regression analyses, it examines linear causal relationships among variables, while simultaneously accounting for measurement error. SEM can be viewed as a combination of factor analysis and regression or path analysis. The interest in SEM is often on theoretical constructs, which are represented by the latent factors that can be interpreted as latent traits or “true” variables (e.g., QoL or distress) underlying the measured items and inducing dependence among them. The measurement model can be of interest in its own right, but the focus of investigation is usually set on the relations among factors or between factors and observed variables (the structural part of the model) (Bollen, 1989, 2002; Browne and Cudeck, 1993). The relationships between the theoretical constructs and/or observed covariates are represented by regression or path coefficients.

Missing data were replaced by way of multiple imputation algorithm. SEM, with a maximum likelihood estimation method, was used to evaluate the fit of the hypothesized model based on the following multiple criteria: χ^2^ test, comparative-fit index (CFI), tucker-lewis index (TLI), coefficient of determination (CD), and root mean square error of approximation (RMSEA). Hypotheses regarding the structural relationships of the constructs in the final model were evaluated using the magnitude of path coefficients (standardized coefficient) and their significance (Bentler, 1990).

## Results

### Participant characteristics

The 261 participants were predominantly male in the clinical sample (56.6%) and female in the control group (57.8%). It was obtained a mean age of 49.1 years (*SD* 9.54 years) in the control group and of 55.5 years (*SD* 13.5 years) in the clinical group. Most of the participants (52.9% in the clinical sample, and 47.2% in the control group) had graduated from high school or secondary school and resulted to be currently married (73.5 and 77.4%). Regarding the employment status, most patients had a full time job: 46.1% of those belonging to the control group and 39.6% of those belonging to the clinical group (Table 1). The clinical sample characteristics are shown in the Table 1. HbA1c level, BMI, and medical complication have been only recorded for the clinical sample. Only 38% of the patients reported a medical complication and the disease duration showed an average of 11.6 years (*SD* 8.9 years).

**Table 1:** **General characteristics of the study sample (***N*** = 261)**.

**Variable**		**Control sample**	**Clinical sample**
Gender	M	42.2%	56.6%
	F	57.8%	43.4%
Education	Elementary school	2.0%	6.3%
	Secondary school	21.6%	47.2%
	High school	52.9%	30.2%
	Bachelor's degree	20.6%	14.5%
	PhD or specialization	2.9%	1.9%
Employment status	Housewife	24.5%	17.6%
	Full time	46.1%	39.6%
	Unemployed	17.6%	7.5%
	Pensioner	9.8%	30.8%
	Student	2.0%	4.4%
Marital status	Never married	16.7%	13.2%
	Currently married	73.5%	77.4%
	Widowed	3.9%	3.1%
	Cohabitant	2.9%	1.9%
	Separated or divorced	2.9%	4.4%
Housing situation	I live alone	4.9%	5.7%
	I live with others	95.1%	94.3%
Age *(M ± SD)*		49.1 ± 9.54	55.5 ± 13.5
Disease duration *(M ± SD)*			11.6 ± 8.9
HbA1c (*M ± SD)*			7.6 ± 1.5
BMI (*M ± SD)*			28.6 ± 5.5
Medical Complications			38.61%

To check the reliability of the emerging factors, Cronbach's α coefficients were computed. The results showed that Cronbach's α (NA, SI, Physical dimension, Psychological dimension, Social Relations, and Environment dimension) resulted greater than 0.70 for all the factors, meaning that each factor scale had consistency.

### Structural equation model results

Using SEM, a comparison was made between the clinical and control sample in order to verify if the latent variables scores, related to DS14 and WHOQOL, had significantly different average levels. The factor loadings estimates for the measurement models used in the comparison are shown in Tables A1 and A2 in the Appendix.

**Figure 1:**
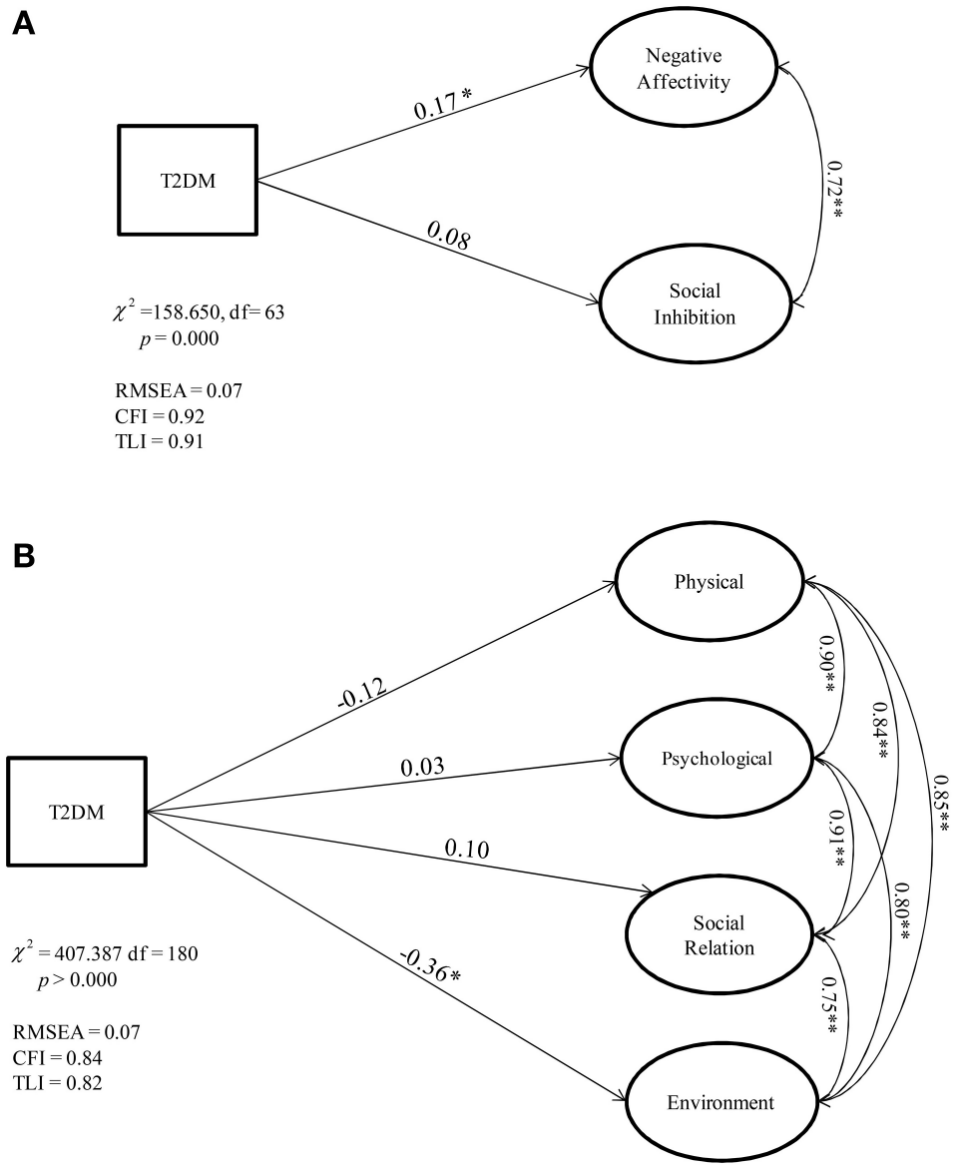
**(A)** Negative Affectivity and Social Inhibition dimensions compared between the control (healthy) and clinical group (with T2DM): Structural Model. **(B)**. WHOQOL dimensions compared between the control (healthy) and clinical group (with T2DM): Structural Model. ^*^p < 0.1, ^**^p < 0.05, ^***^p < 0.01.

Figures 1A,B presents the results of this comparison between the groups. The binary variable T2DM indicated the presence of DM disorder within clinical sample.

A statistically significant difference was found in the clinical sample with higher levels of NA and environmental QoL constructs than controls. More specifically, where NA within clinical sample was higher than control, the environmental QoL of clinical participants was lower than healthy respondents. In other words, patients with T2DM have reported a higher degree of NA and a worse environmental QoL compared to the control group.

**Figure 2:**
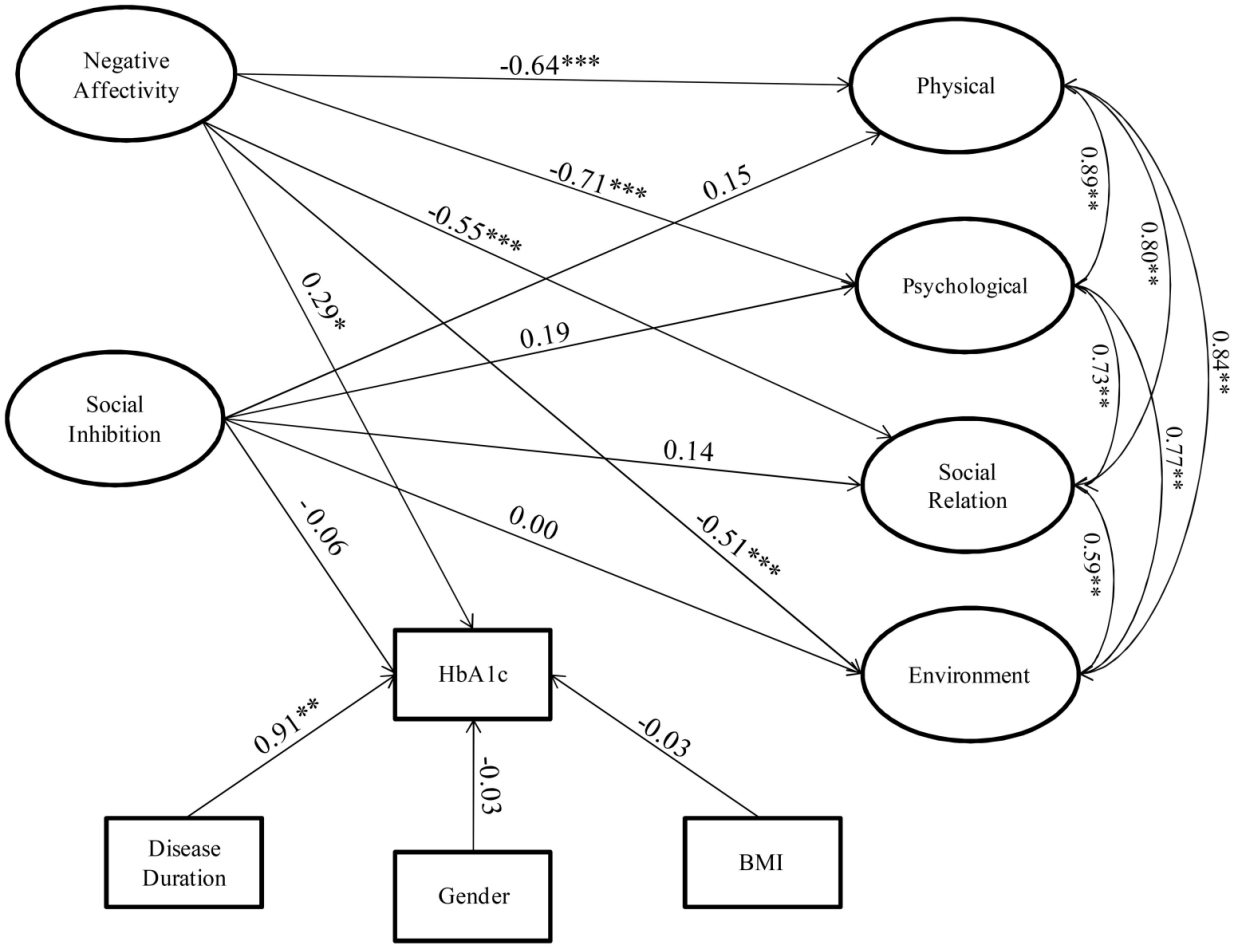
**Relationships among Negative Affectivity and Social Inhibition with WHOQOL dimensions and HbA1c levels in people affected by T2DM: Structural Model Model**. ^*^p < 0.1, ^**^p < 0.05, ^***^p < 0.01.

Data were analyzed, with the aim to verify to what extent the examined psychological factors impact on QoL dimensions and on treatment adherence (HbA1c levels). The structural component of the model included two endogenous latent traits (NA and SI) and four exogenous latent factors for the QoL (Physical, Psychological, Social Relation, Environment). The parameter estimates for the SEM measurement components are provided in the Appendix Tables A3 and A4. The observed exogenous covariates were BMI, gender, and disease duration, while HbA1c was considered as an endogenous observed variable. Figure 2 shows the path diagram and the parameter estimates for the structural model.

The values of multiple fit indices indicated that the proposed model provided a good fit data, χ^2^ = 884.106, df = 482, *p* = 0.00, relative/normed chi-square χ^2^/df = 1.83,TLI = 0.73, CFI = 0.76, CD = 0.96, and RMSEA = 0.06. These values indicate a minimum approximation error, then the model fits the data.

The parameter model estimates indicated that NA exerted significant negative impacts on the physical (−0.64), psychological (−0.71), social (−0.55), and environmental (−0.51) dimensions of the QoL of patients with T2DM. In other words, patients with a larger NA index show a poorer QoL with regards to all domains.

Another result is the effect of the NA on HbA1c: the estimated parameter (0.29) shows that an increasing level of the NA index leads to a growing rate of the level of HbA1c in all the patients affected by DM.

There are no significant direct effects of the SI on QoL and on the level of HbA1c. This is coherent with the finding that there is no significant difference in the mean level of the SI index between the clinical and the control groups, as shown in Figure 1.

The influence of the observed exogenous covariates on the observed endogenous variable (HbA1c) can be detected through the model. In particular, we found a significant effect of disease duration on the glycemic level, while no direct BMI and gender effects were found. In other words, increasing the years of illness leads to an increase of HbA1c.

## Discussion

To the very best of our knowledge, our study is the first original report aiming at the evaluation of the relationship between Type D Personality traits, QoL, and metabolic control in patients with T2DM in the framework SEM. That is, when using SEM as statistics to independently assess NA and SI as specific interdependent constructs contributing to changing levels of QoL and glycemic control in patients with T2DM, we have found a significantly negative impact of NA on all QoL dimensions (i.e., physical, psychological, social relationships, and environmental), as well as a significantly positive impact of NA on HbA1c levels; by contrast, no significant impact between SI and other examined variables has been found. The observed covariates were BMI, gender, and disease duration, while HbA1c was considered an observed variable. In other terms, T2DM patients with the specific NA trait were more likely to show lower rates of glycemic control and QoL.

People with higher NA scores are more prone to experience negative emotions, which include a greater tendency to worry, to experience anger and frustration, guilt, sadness and hopelessness, to perceive self-criticisms, and feel less able to cope with stress. Several studies have discussed the potential physiological mediating mechanisms in the association between personality and T2DM development and progression, including autonomic nervous system activity, HPA-axis functioning, serotonergic dysfunction, platelet activation, and inflammation (Stewart-Knox, 2005; Goldbacher and Matthews, 2007). As an explanation of this association, it is suggested that the development and the exacerbation of T2DM may be the result of a stress-induced cortisol-mediated acute inflammatory immune response (Kiecolt-Glaser et al., 2002; Black, 2003; Seematter et al., 2004). Chronic inflammation has been associated with cardio vascular disease and T2DM, but also with periodontal disease (De Iuliis et al., 2016; Azzi et al., 2017) and arthritis (Crincoli et al., 2016; Vicenti et al., 2016). The finding that depression and anxiety both appear to trigger the release of pro-inflammatory cytokines (proteins that are normally released and signal to other cells in response to infection or injury), provides some support for this argument (Kiecolt-Glaser et al., 2002). Following this hypothesis and analyzing Type D Personality, Mommersteeg et al. (2011) found that the association between Type D personality and change in physical health status over 18 months was partly mediated by inflammatory burden. Earlier writings by Bjorntorp and Rosmond posed that chronic stress affected the development of metabolic syndrome via its effects on the HPA axis (Bjorntorp and Rosmond, 1999). Furthermore, psychological distress leads to a deterioration of positive health behaviors, an increase in deleterious behaviors, and poor compliance with treatment regimens, all associated with insulin resistance (Marshall et al., 1997; Godsland et al., 1998; Mensink et al., 2003). Stronger tendencies to worry and experience other negative emotions may provide decreasing motivation for patients with DM, to follow the necessary self-care regimen and achieve a better clinical outcome. It is easy to understand that medication adherence should have a significant effect on HbA1c level and QoL. Once patients do not adhere to medication, their blood glucose level will inevitably be influenced by the poor medication adherence. The evidence that T2DM patients with NA were more likely to show lower rates of good clinical outcomes is consistent with previously performed studies in T2DM patients with neuroticism (Skaff et al., 2009; Mommersteeg and Pouwer, 2012). However, this is the first time that NA has been examined as a direct predictor of glycemic control and QoL in this clinical population. In a systematic review, Mommersteeg and Pouwer (2012) have reported that a more negative prone personality style is associated with an increased prevalence of DM and its development over time (Mommersteeg and Pouwer, 2012). In a 21-day daily diary study, Skaff et al. (2009) examined the association between negative affectivity and glucose levels. Multilevel analyses revealed a relationship between negative affectivity and glucose levels (Skaff et al., 2009). With respect to SI, we have found no significant impact on all QoL dimensions, as well as no significant impact of SI on HbA1c levels. In our study, we have found/highlighted a significant impact of NA, but not of SI, on healthy outcomes in T2DM population. Our finding has been further confirmed by a study from a sample with acute coronary syndrome, suggesting the primacy of NA over the Type D Personality construct in predicting medication adherence (Molloy et al., 2012). In this study (Molloy et al., 2012), lower levels of self-efficacy could be a mediator between higher levels of NA and poor adherence to medication in patients with coronary heart disease, but no study has showed this primacy of NA in T2DM patients before. The advantage of using the DS14 in patients with chronic medical conditions such as T2DM, is that the items of the scale cannot be confused with somatic symptoms (Nefs et al., 2015), which is a problem with some distress measures such as the Beck Depression Inventory. Such measurement aspect is a clinically relevant clinimetric argument (Bech, 2012) potentially aiming to detect comorbidity. In fact, when evaluating the relative weight (Kissen, 1963) of mental factors on primary medical condition and its outcomes (Tomba and Bech, 2012), somatic and psychological symptoms are differentiated. In addition, the DS14 is a brief instrument and implicates little burden for patients to complete it, allowing for the identification of high-risk patients without the need to apply a more extensive test battery. In other words, when studying the comorbidity between somatic and psychological clinical conditions from a psychosomatic perspective (Fava et al., 2010; Cosci and Fava, 2015), overlapping of items would be avoided by excluding redundant questions. As suggested by Bech (2011), the clinimetrical implication is that the clinical condition can be detected through short but clinically valid rating scales. Hence, several traditional clinical inadequacies linked to the use of very long and boring questionnaires, would be encompassed (Fava et al., 2012). In regards to the medical aspects of our clinical sample, in the model, the effects of covariates on the observed variable (HbA1c) can be detected, where we have found a significant effect of disease duration. In other words, increasing the years of illness also increases the HbA1c. No direct BMI effects have been found on HbA1c. This result can be explained since all patients followed healthy diet, therefore most of them have showed a BMI in the standard. The strengths of the current study include the SEM, a set of statistical techniques, which has allowed a detailed analysis of the impact of NA and SI on glycemic control and QoL through a system of simultaneous equations. Limitations of our study include the use of self-report measures that were not combined with a gold standard as a diagnostic interview, that is, however, what we aim to perform as our main research future perspective. Furthermore, BMI was not detected for the healthy subjects of the control sample, thus this is another limitation of the study.

In conclusion, we have found that, in T2DM population, the specific NA trait not only has a profound negative impact on QoL, but it is also related to worse glycemic control. Therefore, clinically significant variations in personality traits may contribute substantially to the course of medical efforts in DM management. On this basis, a better understanding of the relationship between personality trait and DM treatment could improve DM management. Our data suggest that personality assessment techniques may be clinically useful in identifying individuals at risk for poor glycemic control. This information may be helpful in planning DM education programs for patients who are newly diagnosed, especially if such psychological assessment can identify specific vulnerability personality areas. In addition, research on personality traits in DM may offer new insights that can enhance DM management by combining medical treatment with psychological therapy (Bertini et al., 2014). Finally, NA consists of a specific psychological vulnerability factor affecting medical outcomes of outpatients having a diagnosis of T2DM.

## Author contributions

All authors listed, have made substantial, direct and intellectual contribution to the work, and approved it for publication. Specifically, CC made significant contributions to design the research study, and to draft the article, GD and LF performed the statistical analyses by providing the interpretation of data, and provided significant contributions to draft part of the article, DC and CP provided substantial contributions in drafting part of the article and revising it critically, EV and MF as senior authors gave the final approval of the version of the manuscript to be submitted.

### Conflict of interest statement

The authors declare that the research was conducted in the absence of any commercial or financial relationships that could be construed as a potential conflict of interest.

## Appendix

**Table A1:** **Comparison between the control and the clinical group: Structural Model: factor loading estimates of the measurement model for the DS14 scale**.

**Items**	**Negative affectivity**	**Social inhibition**
(2). I often make a fuss about unimportant things	0.50^**^	
(4) I often feel unhappy	0.70^**^	
(5) I am often irritated	0.75^**^	
(7) I take a gloomy view of things	0.68^**^	
(9) I am often in a bad mood	0.76^**^	
(12) I often find myself worrying about something	0.70^**^	
(13) I am often down in the dumps	0.85^**^	
(1) I make contact easily when I meet people		0.24
(3) I often talk to stranger		0.31
(6) I often feel inhibited in social interactions		0.62^**^
(8) I find it hard to start a conversation		0.70^**^
(10) I am a closed kind of person		0.69^**^
(11) I would rather keep other people at a distance		0.67^**^
(14) When socializing, I don't find the right things to talk about		0.78^**^

**p < 0.1*,

** *p < 0.05*,

*^***^p < 0.01*.

**Table A2:** **Comparison between the control and the clinical group: Structural Model: factor loading estimates of the measurement model for the WHOQOL scale**.

**Items**	**Physical health**	**Psychological**	**Social relationships**	**Environment**
(3) Pain and discomfort	0.38			
(4) Medical treatment	0.51^**^			
(10) Energy	0.74^**^			
(15) Discomfort	0.75^**^			
(16) Sleep	0.45^**^			
(17) Ability to perform daily living activities	0.71^**^			
(18) Capacity for work	0.63^**^			
(5) Positive feelings		0.67^**^		
(6) Self–esteem		0.57^**^		
(7) Thinking, learning, memory and concentration		0.58^**^		
(11) Bodily image and appearance		0.68^**^		
(19) Satisfy with you		0.62^**^		
(26) Negative feelings		0.47^**^		
(20) Personal relationships			0.56^**^	
(21) Social support			0.59^**^	
(22) Sexual activity			0.53^**^	
(8) Freedom, physical safety and security				0.68^**^
(9) Physical environment				0.31^**^
(12) Financial resources				0.63^**^
(13) Opportunities for acquiring new information and skills				0.61^**^
(14) Participation in and opportunities for recreation/leisure				0.61^**^
(23) Home environment				0.56^**^
(24) Health and social care: accessibility and quality				0.23
(25) Transport				0.21

**p < 0.1*,

** *p < 0.05*,

*^***^p < 0.01*.

**Table A3:** **Clinical groups: factor loading estimates of the measurement model for the DS14 scale**.

**Items**	**Negative affectivity**	**Social inhibition**
(2) I often make a fuss about unimportant things	0.47^**^	
(4) I often feel unhappy	0.72^**^	
(5) I am often irritated	0.71^**^	
(7) I take a gloomy view of things	0.61^**^	
(9) I am often in a bad mood	0.68^**^	
(12) I often find myself worrying about something	0.64^**^	
(13) I am often down in the dumps	0.82^**^	
(1) I make contact easily when I meet people		0.18
(3) I often talk to stranger		0.23
(6) I often feel inhibited in social interactions		0.58^**^
(8) I find it hard to start a conversation		0.61^**^
(10) I am a closed kind of person		0.57^**^
(11) I would rather keep other people at a distance		0.66^**^
(14) When socializing, I don't find the right things to talk about		0.56^**^

**p < 0.1*,

** *p < 0.05*,

*^***^p < 0.01*.

**Table A4:** **Clinical groups: factor loading estimates of the measurement model for the WHOQOL scale**.

**Items**	**Physical health**	**Psychological**	**Social relationships**	**Environment**
(3) Pain and discomfort	0.37			
(4) Medical treatment	0.51^**^			
(10) Energy	0.66^**^			
(15) Discomfort	0.71^**^			
(16) Sleep	0.57^**^			
(17) Ability to perform daily living activities	0.73^**^			
(18) Capacity for work	0.68^**^			
(5) Positive feelings		0.66^**^		
(6) Self–esteem		0.56^**^		
(7) Thinking, learning, memory and concentration		0.65^**^		
(11) Bodily image and appearance		0.64^**^		
(19) Satisfy with you		0.68^**^		
(26) Negative feelings		0.48^**^		
(20) Personal relationships			0.59^**^	
(21) Social support			0.53^**^	
(22) Sexual activity			0.49^**^	
(8) Freedom, physical safety and security				0.70^**^
(9) Physical environment				0.35^**^
(12) Financial resources				0.57^**^
(13) Opportunities for acquiring new information and skills				0.59^**^
(14) Participation in and opportunities for recreation/leisure				0.61^**^
(23) Home environment				0.58^**^
(24) Health and social care: accessibility and quality				0.34

**p < 0.1*,

** *p < 0.05*,

*^***^p < 0.01*.
